# **Cardiac screening in pediatric patients with neurofibromatosis type 1: similarities with Noonan syndrome?**

**DOI:** 10.1007/s10554-024-03125-8

**Published:** 2024-05-13

**Authors:** Livia Kapusta, Gil Beer, Ehud Rothschild, Guy Baruch, Gili Barkay, Daphna Marom, Yulia Grinshpun-Cohen, Craig Raskind, Shlomi Constantini, Hagit Toledano-Alhadef

**Affiliations:** 1 Pediatric Cardiology Unit, Faculty of Medicine, Dana-Dwek children’s hospital, Tel Aviv Sourasky Medical Center, Tel Aviv University, Tel Aviv, Israel; 2 Department of Pediatrics, Amalia Children’s Hospital, Radboud University Medical Centre, Nijmegen, The Netherlands; 3 Department of Internal medicine, Tel-Aviv Sourasky Medical Center, Faculty of Medicine, Tel Aviv University, Tel Aviv, Israel; 4 Gilbert Israeli and International Neurofibromatosis Center and the Child Neurology Institute and Child Development Center, Faculty of Medicine, Dana-Dwek Children’s Hospital, Tel Aviv Sourasky Medical Center, Tel Aviv University, 6 Weizmann Street, Tel Aviv, 6423906 Israel; 5 Genetic Institute, Faculty of Medicine, Tel Aviv Sourasky Medical Center, Tel Aviv University, Tel Aviv, Israel; 6 Department of Neonatology, Faculty of Medicine, Tel Aviv Sourasky Medical Center, Tel Aviv University, Tel Aviv, Israel; 7 Department of Pediatric Neurosurgery, The Pediatric Brain Institute, Faculty of Medicine, Dana-Dwek Children’s Hospital, Tel Aviv Sourasky Medical Center, Tel Aviv University, Tel Aviv, Israel; 8 Child Neurology Institute and Child development Center, Faculty of Medicine, Dana-Dwek Children’s Hospital, Tel Aviv Sourasky Medical Center, Tel Aviv University, Tel Aviv, Israel

**Keywords:** Neurofibromatosis, Noonan, Electrocardiography, Echocardiography, Cardiac, Strain

## Abstract

Both Neurofibromatosis type 1 (NF1) and Noonan syndrome (NS) are RASopathies. Characteristic cardiac phenotypes of NS, including specific electrocardiographic changes, pulmonary valve stenosis and hypertrophic cardiomyopathy have not been completely studied in NF1. Purpose: The aims of this study were to assess: (1) similarities in the prevalence and types of ECG and conventional echocardiographic findings described in NS in asymptomatic patients with NF1, and (2) the presence of discrete myocardial dysfunction in NF1 patients using myocardial strain imaging. Methods: Fifty-eight patients with NF1 (ages 0–18 years), and thirty-one age-matched healthy controls underwent cardiac assessment including blood pressure measurements, a 12-lead ECG, and detailed echocardiography. Quantification of cardiac chamber size, mass and function were measured using conventional echocardiography. Myocardial strain parameters were assessed using 2-Dimensional (2D) Speckle tracking echocardiography. Results: Asymptomatic patients with NF1 had normal electrocardiograms, none with the typical ECG patterns described in NS. However, patients with NF1 showed significantly decreased calculated Z scores of the left ventricular internal diameter in diastole and systole, reduced left ventricular mass index and a higher incidence of cardiac abnormal findings, mainly of the mitral valve, in contrast to the frequently described types of cardiac abnormalities in NS. Peak and end systolic global circumferential strain were the only significantly reduced speckle tracking derived myocardial strain parameter. Conclusions: Children with NF1 demonstrated more dissimilarities than similarities in the prevalence and types of ECG and conventional echocardiographic findings described in NS. The role of the abnormal myocardial strain parameter needs to be explored.

Trial registration number: 0212-15-TLV, Date of registration: 06.04.2015.

## Introduction

Neurofibromatosis type 1 (NF1) is a relatively common neurogenetic disorder with birth incidence of 1 in 2,699 individuals [1], [2]. It affects the nervous system, as well as other organs and tissues. NF1 is caused by an autosomal dominant genetic variation in the Neurofibromin gene located on chromosome 17q11.2. This protein is part of the GTPase-activating protein family with tumor suppressor function [3]. Penetrance after childhood is almost complete [4]. There is considerable variation in the clinical phenotype, and specific genotype phenotype correlations were reported, mainly for large deletions and the risk for neurofibromas and tumors [5], [6]. Such genotype phenotype correlations concerning cardiac involvement are rarely reported.

The diagnosis is primarily clinical and [7]confirmatory *NF1* variant is not required in patients fulfilling diagnostic clinical criteria [7]. NF1 syndrome can cause vast multisystem involvement, exact diagnosis and optimal care should be individually designed by multidisciplinary approach [8], [9], [10].

Studies on the prevalence of cardiovascular abnormalities are scarce [5], ranging from congenital heart disease (CHD) to vasculopathy and hypertension, earlier reports suggest that up to 27% of patients with NF1 have a cardiovascular anomaly of different types [11], [12], [13], often without pronounced auscultatory findings. These cardiac anomalies include secundum atrial septal defects, atrial septal aneurysms, peripheral pulmonary artery stenosis, coarctation of the thoracic aorta, dilatation of the aorta, mitral valve prolapse with evidence of trivial mitral regurgitation with myxomatous mitral valve, (sub) aortic valve stenosis, aortic valve regurgitation and pulmonary artery stenosis [5], [11], [12], [13], [14]. Also NF-1 related vasculopathy may present as renovascular hypertension, abdominal aortic coarctation, internal carotid artery aneurysms, or cervical verterbral arteriovenous malformations. Cardiovascular congenital lesions in NF1 although often under-estimated may justify early diagnosis and treatment.

NF1 belongs to a list of various monogenic syndromes, currently grouped together as the RASopathies.These monogenic syndromes are caused by germline mutations in genes that encode components of the Ras/mitogen-activated protein kinase (RAS-MAPK) pathway. Although tradtitionally distinct, they share overlapping phenotypic features including facial dysmorphism, cardiac, cutaneous, musculoskeletal, gastrointestinal and ocular abnormalities, and a predisposition to cancer [15], [16]. Pulmonary valve stenosis and hypertrophic cardiomyopathy (HCM) are common cardiac abnormalites in NS [17], [18]. Those with NS and HCM have a higher risk profile and a worse late survival pattern [19]. Interestingly, the majority of (molecularly confirmed diagnosis) patients with NF1, particularly those with pulmonary valve stenosis, and including half of these patients with other CHDs display NS like features [5]. Cardiomyopaties are also described in NF1, with or without the presence of hypertension [11], [12], [14], [20], [21]. Characteristic electrocardigraphy (ECG) findings have been described in NS, yet they were not associated with a *PTPN11* gene mutation, or with a specific cardiac anomaly. Similar characteristic ECG findings in NF1 were not yet described. Pulmonary stenosis, atrial septal defect and hypertrophic cardiomyopathy are the most comment cardiac abnormalities in NS [18].

The reported prevalence of cardiac abnormalities in patients with NF1 syndrome is likely to be an underestimation because only a small percentage of these patients undergo a proper investigation including echocardiography [11]. There are no recommendations or guidelines for routine cardiac assesments in NF1 patients. Our institution (and many others worldwide) do not screen routinely NF1 patients for cardiac involvement. Myocardial strain imaging is a noninvasive echocardiographic modality to assess global and regional myocardial deformation as a marker of subclinical cardiac disease [22], [23]. Myocardial strain parameters are nowadays recognized as complementary data requested for the early diagnosis of myocardial damage (even in the presence of relatively preserved fractional shortening or ejection fraction), e.g., in monitoring chemotherapy induced cardiotoxicity [24], [25]. No studies on myocardial deformation in patients with NF1, with or without hypertension, were conducted.

The aims of the study were to assess: (1) similarities in the prevalence and types of CHD and ECG changes as described in NS in asymptomatic patients with NF1, and (2) the presence of discrete myocardial dysfunction using myocardial strain imaging.

## Materials and methods

The study was conducted after obtaining approval from the institute’s ethical committee, in accordance with the declaration of Helsinki. The patients and their parents gave written informed consent for publication.

### Study population

Consecutive asymptomatic patients with NF1 aged 0–18 years who visited our Neurofibromatosis tertiary outpatient clinics (IIGNFC) at the Dana Dwek Children’s Hospital during June 2016 and December 2020 were invited to participate in the study. The diagnosis of NF1 was according to the clinical criteria reported by the National Institutes of Health Consensus Statement Conference [7]. Patients were excluded if they had clinical heart failure, a history of cardiovascular disease or chronic renal insufficiency. The patients and/or their guardians gave informed consent, and the study was approved by the local ethics committee.

Patients’ recruitment and clinical data collection was performed at the Neurofibromatosis Outpatient Clinic (IIGNFC). Cardiac evaluation was performed at the Pediatric Cardiology Unit. Patients with NF1 underwent a complete physical examination, blood pressure measurement, a 12-lead ECG and detailed echocardiography. As a control group, we included ECG and echocardiographic studies of healthy age matched children that were routinely referred for echocardiographic evaluation of an asymptomatic, innocent heart murmur or for other screening purposes.

### Echocardiograms

The echocardiograms were performed by an experienced echocardiography technician and supervised by one experienced pediatric cardiologist (LK). Images were obtained with a 8.0 MHz or a 5.0 MHz phased-array transducer depending on the patient’s age and weight, using a locally available ultrasound machine (Siemens 2000, Germany). Quantification of cardiac chamber size, ventricular mass and systolic and diastolic left ventricular function was measured in accordance with the recommendations for chamber quantification by the American Society of Echocardiography’s Guidelines and Standard Committee and the Chamber Quantification Writing Group [26]. An M-mode echocardiogram was performed in the parasternal long- and short-axis views to measure the internal dimensions of the left ventricle at end-diastole (LVIDd) and end-systole (LVIDs), and the posterior and septal wall thickness at end-diastole (LVPWd and IVSd, respectively). Left ventricular mass (LVM) was calculated using the following formula: LVM = 0.8{1.04[ ({LVIDd + IVSd + LVPWd]3 − LVIDd3)]} + 0.6 [27], and was afterwards indexed (LVMI) by the body mass surface area (BSA). Z-scores for LVIDd, LVIDs, IVSd and LVPWd were calculated using an online calculator (parameterz.com) based on a healthy pediatric population [28]. Left ventricular systolic function was determined using fractional shortening (FS), ejection fraction (EF), and rate-corrected velocity of circumferential fiber shortening (VCFc). FS was calculated by the following formula: ((LVIDd-LVIDs)/ LVIDd)/100. Fractional shortening above 27% and EF 53% and higher were considered normal. VCFc was calculated with the formula from Colan et al [29]. The left ventricular diastolic function was evaluated using early (E) and late (A) diastolic trans-mitral peak flow velocity ratio (E/A ratio), deceleration time of early filling velocity (Edec) and isovolumetric relaxation time (IVRT).

### Myocardial strain

Myocardial strain parameters were assessed using 2-Dimensional (2D) Speckle tracking echocardiography image acquisition and off-line analysis. Three subsequent heart beats of apical four-chamber views of the LV and the left atrium (LA) and short axis images of the left ventricle (at the papillary muscle level) were acquired at a frame rate of 60–90 frames/second. All raw datasets were transferred to the core laboratory and the images were analyzed using offline commercial feature-tracking software (2D CPA TomTec Imaging Systems, Germany) by 2 researchers blinded to the patient’s clinical and transthoracic echocardiographic results. The myocardial strain analysis was performed according to previously described methods by our group [30]. In short, one cardiac cycle with optimal image quality was selected. Endomyocardial borders of the LV and LA were automatically traced throughout the cardiac cycle by the software, with manual correction if necessary. For the LV, we evaluated peak and end-systolic global longitudinal strain (LVGLS, average of the basal septum, mid septum, apical septum, apical lateral wall, mid lateral wall, and basal lateral wall), peak and end-systolic GLS of the septal wall, peak and end-systolic GLS of the lateral wall, and peak systolic LV global circumferential strain (LVGCS: average of anterior septum, anterior-, lateral-, posterior-, inferior-, and septal walls at the papillary muscle level). Time to peak (TTP) systolic LVGLS and LVGCS (average of 6 segments, respectively) as well as for the septal and lateral walls (average of 3 segments, respectively) were calculated. All LV strain values are negative values, where a decrease in strain (hence, more positive value) is observed when LV function deteriorates. For the LA we assessed the peak strain in three phases: reservoir (LASr), conduit (LASc) and pump (LASp). LAS values are positive values, where a decrease in LAS (mainly LASr) is observed when LV function deteriorates. In this study, it is important to note that none of the patients evaluated had cardiac tumors that might have affected strain outcome.

### Statistical analysis

The IBM SPSS Statistics 28.0 software was used for statistical analysis. Continuous variables were expressed as means ± standard deviation. Categorical variables were expressed as percentages by using chi-square test for comparison. The comparison between the groups in quantitate variables was conducted by t independent sample t-test (demographic variables and cardiology data). Blood pressure comparison was conducted while covariate age (which is known to affect these indicators). *P* < 0.05 indicated that the difference was statistically significant.

## Results

### Group demographics & clinical manifestations

Two hundred and fifty patients with a clinical diagnosis of NF1 visited the clinic and were offered to participate in the study. Fifty-eight eligible patients consented to participate in the study. Their manifestations of NF1 included: Café au lait macules in 96.6%, freckles in 84.5%, optic pathway glioma in 17.2%, Lisch nodules in 58.6%, cutaneous neurofibroma of plexiform neurofibroma in 27.6%, bone involvement in 17.2%, and an NF1- diagnosed first degree relative in 41.4% of the patients. Confirmatory *NF1* variant was performed in eighteen patients (31%) with NF1.

Demographic characteristics of patients with NF1 and healthy controls are shown in Table 1. No significant differences were noted between the patients with NF1 and the healthy controls in any of these characteristics, including blood pressure values when considering age as a covariate factor.

**Table 1:** Demographic characteristics of patients with NF1 and controls

	Patients with NF1	Controls	*P*-value
Number	58	31	
Age (years)*	8.38 ± 4.06	6.7 ± 4.12	0.067
Gender**	48.3% Male	67.7% Male	0.079
Height (cm)*	125.63 ± 23.92	119.79 ± 26.77	0.295
Weight (kg)*	29.09 ± 15.65	24.56 ± 19.79	0.179
Sys BP (mmHg)***	105.83 (± 12.1)	101.03 (± 12.42)	0.271
Dias BP (mmHg)***	59.67 (± 10.08)	59.49 (± 8.23)	0.507
HR (b/min) *	90.32 ± 17.4	89.03 ± 21.39	0.759

*t-test

***Chi square test

*** Covariate by age

### Electrocardiography

Cardiac function evaluated by ECGs showed a very low prevalence of minor abnormalities in patients with NF1: sporadic atrial (*n* = 1) and ventricular (*n* = 1) premature beats, left axis deviation (*n* = 1) and mild bradycardia (*n* = 1). Minor ECG abnormalities in the controls showed: an incomplete right bundle branch block (IRBBB, *n* = 2) as well as left axis deviation (*n* = 2) and mild bradycardia (*n* = 2, 53 and 55 bpm respectively). All patients and controls showed sinus rhythm with normal conduction durations (PR interval, QRS interval and QTc interval). No ST segment abnormalities were found. Based on ECG, none of the patients met ECG criteria for left ventricular or right ventricular hypertrophy.

### Conventional echocardiography

Quantification of cardiac chamber size, ventricular mass, and systolic and diastolic left ventricular function of patients with NF1 and the healthy controls are shown in Table 2. LV dimensions showed no significant differences between both groups. However, once the Z-scores for the LV dimensions were calculated, both LVIDd (Z) and LVIDs (Z) of the patients with NF1 were significantly decreased, and the LVMI decreased, compared to the healthy controls.

Of fifty-eight patients with NF1, twenty-two (37.9%) had abnormal findings on conventional echocardiography. Cardiac anomalies included: an elongated anterior mitral leaflet with mitral valve regurgitation (MR, *n* = 12), only 2 patients with billowing, patent foramen ovale (PFO, *n* = 4), secundum atrial septal defect (ASD type 2, *n* = 1) with dilated right ventricle that ended in transcatheter closure, mildly dilated left coronary arteries (*n* = 1), pulmonic valve stenosis (PS, *n* = 1), mild dilatation of the main and left pulmonary arteries without pressure gradients ( *n* = 1), patent ductus arteriosus (PDA, *n* = 1), and aortic valve insufficiency (*n* = 1).

**Table 2:** Cardiac dimensions, mass, and function using conventional echocardiography in patients with NF1 and controls

Echocardiographic parameter	Patients with NF1 (*n* = 58) (mean ± SD)	Controls (*n* = 31) (mean ± SD)	*P*-value
IVSd (mm)	5.13 (± 0.98)	4.98 (± 1.16)	0.258
IVSd (Z)	-0.6 (± 0.82)	-0.54 (0.76)	0.369
LVPWd (mm)	4.92 (± 1.13)	4.94 (± 1.27)	0.455
LVPWd (Z)	-0.95 (± 0.78)	-0.76 (± 0.74)	0.143
LVIDd (mm)	37.24 (± 5.08)	37.09(± 6.5)	0.455
LVIDd (Z)	0.053 (± 0.79)	0.52(± 0.81)	**0.003**
LVIDs (mm)	22.19 (± 3.51)	22.51(± 4)	0.35
LVIDs (Z)	-0.49 (± 0.89)	0.036(± 0.85)	**0.004**
LVM (g)	48.25 (± 20.12)	48.91 (± 25.33)	0.446
LVMI (g\$${\text{m}}^{2})$$	48.14 (± 9.36)	52.10 (± 10.31)	**0.035**
FS %	40.35 (± 4.67)	39.08 (± 5.28)	0.12
EF %	71.58 (± 6.84)	70.25 (± 5.51)	0.17
VCFc (%)	44.28 (± 12)	41.79 (± 12.58)	0.189
IVRT (ms)	48.04 (± 10.40)	46.43 (± 13.82)	0.27
MV E/A ratio	1.73 (± 0.37)	1.78 (0.36)	0.275
Edec (ms)	114.66(± 26.45)	11.48(± 23.49)	0.289

IVSd = interventricular septum dimension in diastole ; LV = left ventricular; LVPWd = LV posterior wall dimension in diastole; LVIDd and LVIDs = LV internal dimensions in diastole and systole, respectively; LVM = LV mass; LVMI = LV mass index; FS = fractional shortening; EF = ejection fraction; VCFc = rate-corrected velocity of circumferential fiber shortening; Edec = deceleration time of early filling velocity; IVRT = isovolumetric relaxation time; mm = millimeters; Z = z score; g = grams; m = meter; % = percentages; ms = milliseconds. Statistically significance *p-*values are presented in bold.

### Myocardial strain

2D speckle tracking-derived myocardial strain parameters of patients with NF1 and the controls are shown in Table 3. Neither longitudinal myocardial strain parameters nor the LAS differed between the groups. Peak and end systolic GCS were the only significantly lower parameters in patients with NF1 compared to controls.

**Table 3:** Two-dimensional speckle tracking-derived myocardial strain parameters of patients with NF1 and the controls

Strain parameters	Patients with NF1 (= 58) (mean ± sd)	Controls (*n* = 31) (mean ± sd)	*P*-value
Peak systolic LVGLS (%)	-23.19(± 3.68)	-22.91 (± 3.22)	0.359
ES- LVGLS (%)	-22.98 (± 3.67)	-22.73 (± 3.28)	0.378
TTP systolic LVGLS (ms)	293.53 (± 38.12)	289.52 (± 33.48)	0.311
Peak systolic septal wall strain (%)	-22.88 (± 3.62)	-22.77 (± 4.02)	0.447
ES septal wall strain (%)	-22.59 (± 3.62)	-22.43 (± 4.2)	0.426
TTP systolic septal wall strain (ms)	293.97(± 38.77)	285.55 (± 32.63)	0.153
Peak systolic lateral wall strain (%)	-23.73(± 4.12)	-22.56(± 2.86)	0.081
ES lateral wall strain (%)	-23.29 (± 4.43)	-22.43 (2.85)	0.164
TTP lateral wall strain (ms)	297.5 (± 34.34)	296.16 (± 33.74)	0.43
Peak systolic LVGCS (%)	-31.62 (± 7.31)	-34.11 (± 3.91)	**0.019**
ES-LVGCS (%)	-31.31 (± 7.36)	-33.86 (± 3.94)	**0.018**
TTP-LVGCS	299.14 (± 34.61)	297.1 (± 47.64)	0.232
LASr, (%)	62.26 (± 9.65)	60.59 (± 9.7)	0.47
LASc, (%)	47.69 (± 8.91)	47.56 (± 7.87)	0.96
LASp (%)	14.57 (± 5.89)	13.03 (± 6.46)	0.29

LVGLS = left ventricular global longitudinal strain; ES = end systolic; TTP = time to peak; LVGCS = left ventricular global circumferential strain; LASr = left atrium strain reservoir; LASc = left atrium strain conduit; LASp = left atrium strain pump; ms = milliseconds; (%) = percentages. Statistically significance *p-*values are presented in bold

## Discussion

This study is the first to attempt to identify in NF1 patients without known syndromic complications the prevalence and types of ECG changes and cardiac abnormalities as described in NS. Patients with NF1 had normal electrocardiograms, none with the typical ECG patterns described in NS. However, patients with NF1 showed significantly decreased LVIDd (Z) and LVIDs (Z), reduced LVMI and a higher incidence of cardiac abnormalities, mainly of the mitral valve, in contrast to the frequently described types of cardiac abnormalities in NS. LVGCS was the only significantly reduced speckle tracking derived myocardial strain parameter.

Hypertension is also common in pediatric patients with NF1 [31]. Hypertensive patients present thicker myocardial walls, an increased LVMI. Our pediatric patients were young and showed normal systolic and diastolic blood pressures (covariate by age) during their visit to the outpatient clinics. They did not have thicker myocardial walls or increased LVMI. However, hypertension might still develop later in life.

Cardiac function evaluated by the ECG showed a very low prevalence of abnormalities in both patients with NF1 and in controls. NF1 syndrome and NS are both considered as part of the RASopathies syndromes. In patients with NS, the ECG showed at least one characteristic finding in 50% of cases including left axis deviation, small R waves in the left precordial leads and an abnormal Q wave. These ECG patterns were not associated with a PTPN11 gene mutation, or with a (specific) cardiac anomaly [18]. In this study, only one patient with NF1 showed left axis deviation. None had ECG alterations except for clinically insignificant mild bradycardia and sporadic premature beats.

Neurofibromin has crucial involvement in the cardiovascular system in NF1.It modulate epithelial-mesenchymal transformation and proliferation in the developing heart [32]. Activation of pathways known to be involved in cardiac hypertrophy and dysfunction are seen with the loss of myocardial neurofibromin. The Ras family of small Guanosine Triphosphate (GTP)-binding proteins (G proteins) represents one of the main components of intracellular signal transduction required for normal cardiac growth but is also critically involved in the development of cardiac hypertrophy and heart failure [25]. The central role of Ras in pathologic and physiologic cardiac hypertrophy has been demonstrated in multiple in vitro and in vivo settings [25]. HCM and dilated cardiomyopathy (DCM) are the known cardiomyopathies in NS. But, the cardiomyopathies in NF1 can be distinct and might be related to the role of the neurofibromin protein in cardiac development, as well as perhaps underlying coronary vascular abnormalities [33].

As reported previously in patients with NS, pulmonary stenosis was the most common CHD, followed by atrial septal defect and hypertrophic cardiomyopathy [17], [18]. In our study of patients with NF1 only one patient had PS, and a second had secundum ASD. No hypertrophic cardiomyopathy was found in our patients or in those of Incecik [13] and Lin [11]. Compared to healthy controls, the conventional echocardiographic parameters showed a significantly decreased Z- scores of LV internal diameters, yet without decreased systolic and diastolic function. Also, the LVMI was already reduced, in contrast to patients with NS. These findings do not match the disposition of HCM which is frequently observed in RASopathies. Other cardiac abnormalities were found in 37.9% of our patient group, which is a higher incidence of cardiac abnormalities than previously reported [12], [13]. The main cardiac anomalies identified in our study were 12 unknown mitral valve regurgitations, 2 of whom had an elongated anterior mitral leaflet with billowing. Interestingly, a high prevalence of mitral valve anomalies was also previously reported by Pinna et al. [5], but with overall different types, including prolapse, thickening and dysplasia of the valve. It has been shown that cardiovascular malformations were noted more often in patients with large gene deletions [12], [20], [34]. However, the underlying mechanisms of phenotypic heterogeneity of NF1 may be related to the impact of NF1 gene mutations and genetic modifiers [6]. The latter may suggest a delay in onset and development of different manifestations of NF1.

Myocardial strain parameters are nowadays seen as complementary to conventional echocardiography in the assessment of cardiac function [24]. Furthermore, recent studies demonstrate the ability of myocardial velocities and deformation to detect abnormalities related to genetic disease [35], [36] In a recent study of Caiffa et al. on a very limited number of NF-1 patients (*n* = 17) treated with Selumetinib, subtle changes were identified in GLS compared to healthy controls. However, our study analyzed asymptomatic NF1 patients without any treatment [37]. When using 2D Speckle tracking echocardiography, the strain parameters demonstrate decreased LVGCS in patients with NF1 as compared to controls. This may suggest the presence of discrete myocardial dysfunction at the fiber muscle level. GCS measurement may provide useful insight into cardiac dysfunction mechanisms, since it contributes more to LVEF, than GLS [38] However, varying normality thresholds in literature hamper a clinically useful definition of abnormality. To assess the role of these newly described parameters in predicting cardiovascular outcome of pediatric patients with NF1 one needs to continue to follow-up this cohort and enroll new patients for future clinical outcomes studies. Furthermore, the correlation of NF1 mutations and micro-deletions with abnormal global and regional myocardial deformation indices needs to be further explored.

In the current study, only 8 (36.4%) out of the 22 patients with CHD underwent molecular testing of the NF1 gene. Furthermore, no specific genetic variation was more frequent in the group of 18 genetically tested patients (with or without CHD), which is in concordance with previous reports [5]. Further delineation of the genetic variations in children with NF1 and cardiac involvement is mandatory.

### Limitations

Our study has several limitations. It is a single center study with the main limitation of a relatively small patient sample size and a small number of healthy controls. Furthermore, the development of impairments in cardiac function over time was not evaluated.

This is a preliminary study evaluating the prevalence of LVGLS, LVGCS and LAS in patients with NF1. Strain analyses require time and expertise. Many vendors implement semi-automated measurements to expedite its clinical use. To assess the role of these newly described parameters in predicting cardiovascular outcome one needs to continue to follow-up this cohort and enroll new patients for future clinical outcomes studies.

The small number of patients with NF1 who underwent molecular testing of their NF1 gene prevents further research on the correlation of NF1 mutations and micro-deletions with abnormal conventional and myocardial deformation parameters.

## Conclusion

Asymptomatic pediatric patients with NF1 demonstrated dissimilarities, rather than similarities, in the prevalence and types of ECG and echocardiographic changes as described in Noonan syndrome. The patients presented a high incidence of cardiac abnormalities, mainly of the mitral valve. The decreased calculated Z-scores the of LV internal dimensions as well as reduced LVMI, and the presence of discrete myocardial dysfunction using myocardial strain imaging should be further evaluated over time.

## Data Availability

The data from this study are available on reasonable request from the corresponding author.
